# Intracoronary Diagnostics in Patients with Acute Coronary Syndrome

**DOI:** 10.31083/j.rcm2402045

**Published:** 2023-02-06

**Authors:** Qianhui Sun, Minghao Liu, Ming Zeng, Haibo Jia

**Affiliations:** ^1^Department of Cardiology, The Second Affiliated Hospital of Harbin Medical University, 150086 Harbin, Heilongjiang, China; ^2^Key Laboratory of Myocardial Ischemia, Harbin Medical University, 150086 Harbin, Heilongjiang, China

**Keywords:** acute coronary syndrome, intracoronary imaging, coronary physiology

## Abstract

Despite the increasing number of coronary interventions in China, long-term adverse cardiovascular events remain high, especially in patients with acute coronary syndromes (ACS). The advent of intracoronary imaging and coronary physiological diagnostic techniques, such as optical coherence tomography (OCT), intravascular ultrasound (IVUS), near infrared spectroscopy (NIRS), and flow reserve fraction (FFR), has optimized the diagnosis and risk classification of patients with ACS. Intracoronary diagnostics compensate for the deficiencies of conventional coronary angiography in identifying and incriminating lesions and high-risk lesions. The combination of intracoronary imaging and physiological techniques is expected to achieve a comprehensive evaluation of the structural features and physiology of the coronary arteries, thus further tailoring and improving the prognosis of patients.

## 1. Overview of Acute Coronary Syndrome 

Coronary heart disease (CHD) is the most common clinical manifestation of atherosclerosis. Acute coronary syndrome (ACS), considered the most severe type of CHD, poses a serious threat to human life [1]. ACS usually includes ST-segment elevation myocardial infarction (STEMI), non-ST-segment elevation myocardial infarction (NSTEMI) and unstable angina pectoris. Among these, myocardial infarction is typically due to myocardial cell necrosis caused by prolonged ischemia [2]. Coronary atherosclerosis is a major cause of ACS. Atherosclerosis is usually considered to be the formation of a thrombus blocking the lumen area due to injured vascular endothelial lesions, which leads to a sharp decrease in coronary blood flow. In recent years, the prognosis of ACS patients has dramatically improved with the development of percutaneous coronary intervention (PCI) and cardiovascular medicine. Coronary angiography used to be the gold standard for diagnosing CHD. Nevertheless, like traditional two-dimensional imaging, it has certain limitations. The inability to accurately evaluate the pathophysiological mechanism, plaque burden (PB), plaque characteristics, and stent lesions, may lead to misjudgment of lesion characteristics and suboptimal treatment.

The 2018 ESC/EACTS guidelines emphasize the importance of using intracoronary imaging techniques to aid in the precise diagnosis of patients with myocardial infarction [3]. For culprit lesions, the current guidelines point out that treatment decisions should be made based on lesion types and characteristics, such as considering conservative treatment for plaque erosion and rotational atherectomy for large calcified nodules. Such treatments may reduce the number of stents implantation and the incidence of related complications without affecting long-term outcomes [3, 4].

In addition to treating culprit lesions, the treatment of non-culprit lesions is also an essential step in improving the prognosis of patients. Several clinical trials have shown that over half of ACS patients have non-culprit lesions [5, 6, 7] that may develop later and lead to new adverse events. Accordingly, we must also pay attention to these non-culprit lesions and further explore suitable treatment strategies. Intracoronary imaging modalities enable the operator to have a deeper understanding of the internal structure and lesion characteristics of coronary arteries in ACS patients and to optimize the selection of diagnosis and treatment schemes [8, 9]. For ACS patients, intracoronary imaging and physiological technology have become increasingly important in optimizing the comprehensive treatment of culprit and non-culprit lesions.

## 2. Optimization of Diagnosis and Treatment of Patients with Coronary Heart Disease by Intracoronary Examination

At present, there are two main types of intracoronary examination methods. The first involves intracoronary imaging technology, including optical coherence tomography (OCT), coronary intravascular ultrasound (IVUS), and near-infrared spectroscopy (NIRS). The second entails coronary physiological evaluation, such as fractional flow reserve (FFR). Compared to angiography, intracoronary imaging techniques can provide more information on the structural characteristics of plaques with higher resolution and tissue differentiation. Currently, they are the best means for assessing plaque morphological characteristics.

OCT is based on depth-resolved infrared reflection, with an axial resolution of about 10–15 μm and penetration depth of 0.1–2.0 mm. OCT can clearly distinguish plaque composition and structure [10]. However, OCT requires complete blood clearance from the lumen for high-quality imaging since red blood cells cause scattering of the light. IVUS is another common intracoronary imaging technology used to observe coronary plaque. Introduced by Yock *et al*. [11] in the 1980s, IVUS is the first real-time and high-resolution intracoronary imaging device. Its imaging is primarily carried out by collecting and processing acoustic signals. Although the axial resolution of IVUS is about 150 mm, which is less than that of OCT, its penetration depth can reach 8.0–10.0 mm. IVUS thus allows real-time tomographic assessment of the deep contents of plaque and the full-thickness structure of the blood vessel wall, such as media and adventitia; it also permits evaluation of the vascular remodeling. These features play a more significant role in guiding the treatment of coronary diseases in large lumens, such as the left main blood vessel [12, 13]. NIRS is a spectrum-based analysis that has high accuracy in identifying lipid and plaque components by using different optical reflections on tissues. In human experiments, the accuracy and specificity of NIRS in identifying lipid components are over 90% [14].

### 2.1 Accurate Identification of Culprit Plaque by Intracoronary Imaging Tools

For patients with typical acute chest pain or accompanying ST-segment elevation of electrocardiogram, intracoronary imaging can accurately delineate the continuity of vascular lumen, plaque disruption, and associated thrombus to determine the culprit lesion [15]. The most common cause of ACS events is coronary atherosclerosis, whereby the normal triple-layered structure of the vessel wall is absent on the intracoronary image picture. OCT, with its superior resolution, permits clearer visualization of plaque-specific features. Its accuracy has been well demonstrated in correlation with histology [16]. In OCT images, the fibrous component usually appears as a highly expressed region of uniform signal. The lipid component in OCT usually appears as a low-signal region with diffuse boundaries [17, 18]. However, due to the strong attenuation of the lipid component to the light signal, it is generally difficult to observe the posterior border of the lipid and the deep tissue components.

Plaque rupture is the most frequently observed substrate for ACS, accounting for approximately 60%–80% of cases. Defined as the discontinuity of the fibrous cap, it leads the underlying necrotic core components, such as lipids, to communicate with the lumen. Typically, a signal-free cavity inside the plaque can be seen by OCT or IVUS at the fibrous cap rupture, where the necrotic core is washed away by blood flow or optical contrast media. These components promote the formation of (generally red) thrombus, which is rich in red blood cells and leads to a sharp decrease in the lumen area [19]. In this context, thrombosis and vasoconstriction may lead to acute cessation of the coronary blood flow and subsequent myocardial ischemia [20]. Nowadays, clinical treatment tends to recommend stent treatment for most ruptured plaques. Compared with the other type of plaque, plaque rupture has more lipid components and poorer prognosis [21].

The second most common lesion type is plaque erosion, accounting for about 30% of cases. Characterized by an intact fibrous cap, it is typically accompanied by the formation of local platelet-rich white thrombus, blocking blood vessels and is usually attributed to the attenuation of vascular endothelial cells [22, 23]. However, although the endothelial cells are undetectable by current imaging modalities, OCT is the only tool that may identify plaque erosion with its high resolution in clinical settings. Jia *et al*. [24] preliminarily determined the diagnostic criteria of plaque erosion by detection of 126 ACS patients on OCT: A “definite” plaque erosion is described as the absence of fibrous cap disruption, in a lesion frequently composed of fibrous tissue with overlying luminal white thrombus. A “possible” OCT-plaque erosion is defined as an irregular luminal surface without evident thrombus or an overlying thrombus with attenuation of the underlying plaque, without evidence of superficial lipid or calcification in the vessel upstream or downstream of the thrombus site [24].

Several studies of plaque erosion by OCT have demonstrated that the clinical characteristics of plaque erosion patients usually present with the characteristics of young age (<55 years old), smoking, female, and often present in the form of NSTEMI [24]. Erosion plaque is usually characterized by less stenosis, a thicker fibrous cap, and shorter plaque length [24]. Some specialist suggests that such patients may achieve a better prognosis with less severe vessels compared to those with ruptured plaques [25]. In addition, by observing the pathophysiological mechanism of plaque erosion, some studies suggest that a conservative non-stenting strategy may achieve a better prognosis than traditional stents for some plaque erosion with large lumen area and residual diameter stenosis less than 70% [15, 26, 27, 28]. According to the latest EROSION III study comparing angiography and OCT-guided PCI treatment, the number of stents implanted was reduced in the OCT group compared to angiography (15%), while similar prognosis was shown in both groups [4]. This trial again demonstrated the safety of non-stenting strategy and the importance of OCT for optimizing PCI strategies.

The calcified nodule is usually associated with a calcified plate with fibrous cap disrupture, overlaid by a thrombus. When the calcification is thin enough for light to penetrate, OCT can accurately identify the boundary and angle of calcification with sharp borders presenting with signal-poor regions. Calcification can be divided into three types under OCT: eruptive calcified nodules, superficial calcific sheet, and calcified protrusion. Superficial calcification, the most frequent type, often occurs in the left anterior descending coronary arteries, and is likely associated with the most significant post-intervention myocardial damage [29]. Although calcified nodules are relatively rare compared with the other two types, it can pose significant challenges for selecting stent sites and proper stent implantation, like heavy local calcification may lead to poor stent expansion. The factors affecting the adverse effects of stent expansion include lesions with calcium deposit with maximum angle >180°, maximum thickness >0.5 mm, and length >5 mm [30], suggesting that adequate pre-treatment should be given due attention. Previous IVUS studies have also pointed out that superficial calcium angle >270°, longer than 5 mm, 360° of superficial calcium, calcified nodule, and vessel diameter <3.5 mm are all independently related to poor stent expansion rate [31]. Accurate identification of calcification types is beneficial to individualized guidance of stent placement and improvement of the prognosis [32].

Non-obstructive myocardial infarction (MINOCA) is also one of the causes of ACS, typically presenting with less than 50% coronary stenosis. Previous studies have pointed out that MINOCA patients’ 12-month mortality rate can reach 4.7%, much higher than that of the non-ACS population [33]. Another study also noted greater mortality from non-cardiac causes during one-year follow-up in the MINOCA population compared to the NSTEMI population [34]. Common causes of MINOCA include plaque rupture, plaque erosion, coronary artery spasm (CAS), coronary microvascular spasm, and spontaneous artery coronary dissection [2]. CAS is an important etiology of MINOCA, as spasm causes vasoconstriction and subsequent epicardial or transmural myocardial ischemia with transient ST-segment changes. Usually, CAS can be confirmed by provocation test. However, some researchers have disputed the prognostic significance of provocation test in ACS [35], suggesting that it may risk potential arrhythmias or other adverse event in acute stage. Therefore, provocation test is not currently recommended for clinical use. However, a trial exploring patients with AMI by using this test obtained the opposite result [36]. The results of an intraoperative acetylcholine and ergometrine provocation test combining OCT suggest that patients with positive result had a poorer prognosis and were mainly characterized by major adverse cardiovascular events (MACE), especially death, ACS or revascularization [36]. This trial confirms the safety and efficacy of OCT combined with acetylcholine and ergometrine provocation test for intracoronary diagnosis of CAS and identification of MINOCA etiology. Notably, although nonspecific vasodilators are the current standard of care for CAS, the clinical prognosis of the patients in this trial was still suboptimal, possibly owing to the limited current pharmacological treatment options and the fact that most of the patients had drug-refractory angina [37, 38].

In addition, it has been suggested that abnormal microcirculatory function may be a possible important cause of MINOCA [39, 40]. Microvascular overreaction to vasoconstrictors can also lead to myocardial ischemia. The common invasive procedures used to assess microvascular function include (1) impaired endothelial non-dependent function, as measured by coronary flow reserve (CFR) and index microvascular resistance (IMR), and (2) endothelium-dependent function, which need the use of pharmacological stimuli to induce [41]. Among these, CFR refers to the maximum increase in coronary blood flow above resting values after coronary vasodilation, reflecting the combined vasodilatory capacity of epicardial and microvascular coronary arteries. CFR should be assessed together with FFR (response to the degree of epicardial stenosis) when used alone to assess microvessels. Two randomized clinical trials are currently investigating whether customized drug therapy for MINOCA based on the results of adjuvant invasive testing can improve prognosis (NCT05198791 and NCT05122780).

Intracoronary imaging tools, especially OCT, helps to observe thrombosis without prominent atherosclerotic plaque or to find possible thromboembolism or vasospasm and other non-atherosclerotic lesions. Therefore, for patients with atypical ACS, intracoronary imaging is helpful for accurate diagnosis of ACS [42], as it can identify culprit and non-culprit lesions and avoid unnecessary exposure of antiplatelet drugs and anticoagulants.

### 2.2 Accurate Identification of Vulnerable Non-Culprit Lesions by Intracoronary Imaging Tools

As mentioned, the intracoronary imaging tool can delineate the surface structure characteristics of plaque, microchannels, cholesterol crystals, and other important related data [43]. It can divide plaque into vulnerable and non-vulnerable portions by identifying the attributes of non-culprit lesions. It is generally believed that the recurrent adverse events in some patients with ACS after an operation are secondary to vulnerable plaques. In other words, plaques with a large lipid core, thin fibrous cap, and rich macrophages are prone to progress rapidly in a short time or even lead to events, including cardiac death during short-term and long-term follow-up. Moreover, vulnerable plaques in ACS patients usually reside in segments with tight stenosis in the epicardial coronary arteries [44]. In addition, an experiment using OCT specifically to explore ACS patients obtained similar conclusions: Plaques equipped with lipid plaque (maximum lipid angle >180°) and thin fibrous cap plaque (thinnest fibrous cap thickness <65 μm) have a higher risk [45]. It has been demonstrated that in patients with STEMI, those containing non-culprit leisons characterized by thin fibrous cap thickness (TCFA) (lipid angle >90°, mean fibrous cap thickness <65 μm) have more severe lesions [46].

Moreover, IVUS has been also used to study vulnerable plaques *in vivo* for the last ten years. The PROSPECT study, including 697 ACS patients, is a large-scale prospective multicenter trial. After a median follow-up of 3.4 years, it confirmed that the thin fibrous cap plaque, PB ≥70%, and minimum lumen area (MLA) ≤4.0 ${\mathrm{mm}}^{2}$ observed by virtual histology-intravascular ultrasound (VH-IVUS) are closely related to the poor prognosis of patients [7]. This result showed that about one-fifth of non-culprit lesions are closely related to recurrent events, emphasizing the importance of early identification of high-risk lesions. Although the lateral resolution of IVUS is far inferior to that of OCT, the recently developed 60-MHz high-resolution intravascular ultrasound (HR-IVUS), as a new generation of IVUS, has dramatically improved its sensitivity in plaque recognition [47].

The lipid core burden index (LCBI) was the main index for evaluating vulnerable plaques by NIRS. Max LCBI4mm was more commonly used [48, 49]. Schuurman *et al*. [50] use NIRS and IVUS to predict high-risk plaque progressing to recurrent events which includes 117 ACS patients. The results show that with an increase of 100 units, the incidence of MACE increased by 19% [50], underscoring the crucial predictive role of lipid load in adverse prognosis.

### 2.3 Joint Recognition of Vulnerable Plaque by Multimodality Imaging Tools 

**Fig. 1.**
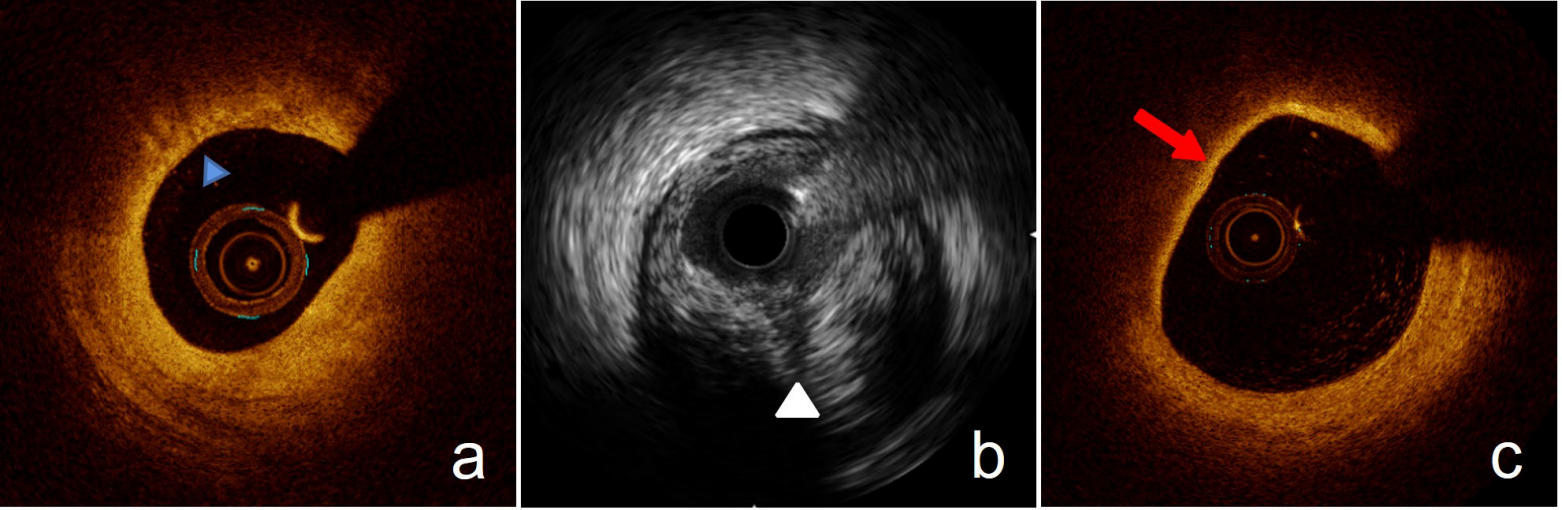
**Common characteristics of vulnerable plaques on intracoronary imaging images**. (a) MLA = 2.51 ${\mathrm{mm}}^{2}$ and macrophages (blue triangle) under OCT. (b) Low backscattering (white triangle) indicating PB >70% under IVUS. (c) A thin fibrous cap plaque delineating necrotic core with an overlying fibrous cap (red arrow) where the minimum thickness of the fibrous cap is less than 65 μm defined by OCT.

In addition to the aforementioned research on single intracoronary imaging technology, the ATHEROREMO-IVUS study uses the combination of IVUS-NIRS to demonstrate that TCFA defined by IVUS is related to the incidence of long-term (more than six months) ACS events. The short-term prognosis of patients will be affected by both PB ≥70% and TCFA [51]. The average follow-up results of 4.7 years show that MLA and TCFA (and PB ≥70%) can independently predict the adverse prognosis of patients [52]. The PROSPECT2 study also redefined vulnerable plaque by combining NIRS with IVUS. Based on the original definition, it added max LCBI4mm ≥324.7 as a new standard to describe vulnerable plaque, optimizing the patient risk classification [53]. The study also suggests that lesions with large plaque burden and high lipid load index are associated with future undesirable cardiovascular events. It indicates that early interventional therapy for vulnerable plaques may further achieve better outcomes. By identifying vulnerable plaques and people with high-risk characteristics via intracoronary imaging technology, targeted treatment may be realized, such as shortening follow-up intervals and strengthening drug therapy. However, this superiority still needs to be confirmed by numerous large-scale trials in this field. Moreover, the combination of OCT and IVUS was also used to explore the pathological features of ACS patients [54, 55]. The various effects of these vulnerable characteristic are listed in Table 1 (Ref. [51, 52, 53, 54, 55]) and Fig. 1, respectively.

**Table 1.** **The summary of studies illustrating vulnerable plaques identified by intracoronary imaging in ACS patients**.

Study	Technology	Vulnerable characteristic	Reference
ATHEROREMO-IVUS	IVUS-NIRS	IVUS virtual histology-derived TCFA; PB ≥70%	[51, 52]
PROSPECT2	IVUS-NIRS	Max LCBI4mm ≥324.7; PB ≥70%	[53]
Wenbin Zhang *et al*.	IVUS-OCT	ACS presentation was related to plaque vulnerability (more TCFA, more lipid and macrophages, larger PB and positive remodeling)	[54]
Francesco Prati *et al*.	IVUS-OCT	MLA <4 ${\mathrm{mm}}^{2}$, FCT <75 μm predicts acute events	[55]

PB, Plaque Burden.

### 2.4 Dynamic Evaluation of Plaque Changes with Intracoronary Imaging Tools 

In predicting the prognosis of patients, it may not be possible to precisely reflect the progression of plaque with only single, one-time points, since the progression is a dynamic process. Measuring OCT at different time points can delineate the vascular condition at different time points. Researchers can use markers such as side branches, calcification, and stent edges to identify the exact sites through different time points to obtain the continuous changes of plaque. Therefore, some studies evaluate plaque response to drugs or plaque progression through more than one time OCT measurement [56, 57]. For example, the HUYGENS study evaluates the effect of proprotein convertase subtilisin/kexin type 9 (PCSK9) inhibitors on plaque by observing the change in fibrous cap thickness [56].

Notably, although OCT can observe the microstructure in the lumen, it cannot calculate plaque depth and area well because of its limited penetration depth. Multiple IVUS measurements are also used clinically to observe plaque change [58]. Some experiments combined IVUS and OCT show that statins can lead to plaque morphological changes, namely, fibrous cap thickening, plaque volume increase [59] and a reduced prevalence of ruptured plaque [60]. The recently released result of the PACMAN AMI study comprehensively evaluated the entire plaque of acute myocardial infarction (AMI) patients by combining IVUS-NIRS and OCT [61]. It observed the continuous composition and morphological changes of plaque *in vivo * [61]. These experiment not only enhanced the credibility of the conclusions but also paved a new way for accurate evaluation and individualized treatment of plaque by combining the advantages and overcoming the disadvantages of three imaging methods.

## 3. Application of Coronary Physiological Tools in ACS

The concept of pressure-derived FFR was first introduced by Pijls in 1995 [62], and has been used in PCI guidance of simple and complex multi-vessel diffuse lesions in clinical practice. FFR was calculated as the ratio of mean distal coronary artery pressure (Pd) to proximal coronary artery pressure (Pd/Pa) by injecting intracoronary adenosine under the condition of maximum myocardial filling. Increasing evidence shows the safety and effectiveness of FFR in guiding the treatment of non-culprit lesions [63, 64, 65]. Generally, FFR >0.8 indicates FFR-negative lesion; FFR <0.75 indicates positive; while 0.75 < FFR < 0.8 is the gray area of measurement [3].

Using FFR to guide the treatment of ACS patients can carry out targeted operations on the lesion areas with blood flow restriction (including culprit lesion and non-culprit lesions). The DANAMI-3-PRIMULTI study, by comparing FFR-guided lesion revascularization and complete revascularization in STEMI patients separately, noted that there was no statistical difference in all-cause mortality and non-fatal revascularization event rates between the groups. However, the complete revascularization group had a lower recurrence and lower probability of revascularization (both urgent and non-urgent) within two years after operation [66]. Similar findings were obtained in several studies, confirming the importance of complete revascularization [67]. These trials also emphasize the importance of treating non-culprit lesions with blood flow restriction in ACS patients. Besides, the advantages of complete revascularization compared to revascularization for culprit lesions only are not only reflected in the reduced number of subsequent revascularization lesions but also the compound outcome of long-term cardiovascular death or myocardial infarction [68].

Recently, FLAVOUR study [69] compared the interventional therapy of patients with moderate stenosis guided by FFR and IVUS. After 24 months of follow-up, FFR guidance was not inferior to IVUS guidance in combined major outcomes of death, myocardial infarction, or revascularization [69]. What’s more, FFR-guided PCI and stent implantation strategies have also achieved better results in patients with ACS. A subgroup analysis of the FAME study confirms these findings through the secondary analysis of 325 NSTEMI and UA. The incidence of MACE after two years in PCI patients guided by FFR among these patients (5.1%) has no obvious difference compared with stable angina pectoris (SAP) (3.7%). In addition, FFR-guided procedures are less time-consuming than angiography, reducing patient exposure to contrast media and radiation [70]. The FUTURE study pointed out that FFR-guided PCI can reduce the proportion of lesions requiring revascularization without increasing the risk of ischemic cardiovascular events or death, thus reducing the economic burden of patients while reducing the risk of stent complications [71]. A prospective multicenter study on NSTEMI patients also pointed out that FFR-guided revascularization therapy has a higher proportion of conservative treatment without increasing the proportion of MACE, including cardiac death, hospitalization for myocardial infarction or heart failure [72]. Hospitalization time and economic burden are also reduced [73]. In addition, an experiment on 304,548 ACS patients to compare FFR-guided PCI with angiography-guided PCI showed that FFR-guided patients had less all-cause mortality and fewer complications such as bleeding and coronary dissection [74].

However, the routine use of FFR in ACS patients remains controversial [75]. Several studies note that FFR-guided revascularization did not show better results than the angiography-guided group [76]. Other studies also point out that compared with patients with SAP, ACS patients with delayed treatment of non-culprit lesions guided by FFR have a poorer prognosis and higher incidence of MACE [77, 78]. The main reasons are as follows: (1) in the acute stage, the increase of adrenaline secretion may lead to excessive contraction of peripheral blood vessels and microcirculation. (2) The microvascular dysfunction caused by myocardial ischemia is not limited to the myocardium attached to the culprit artery, resulting in a false negative (FFR >0.8) [79, 80]. Since measuring FFR in acute stage may underestimate the severity of ischemia, it is still necessary to surveil patients with negative FFR. (3) Some studies have shown that the FFR value of most non-culprit lesions is mainly concentrated in the “grey value” period of 0.75–0.80 [81, 82]. As the treatment method cannot be determined by cutoff value alone, the accuracy of clinical diagnosis of FFR in ACS patients in the acute stage decreases accordingly. The physiological assessment of non-culprit lesions in acute stage by FFR will also underestimate the degree of stenosis to a certain extent, thus changing the treatment strategy. (4) FFR measurement during the initial operation may prolong the time of reperfusion, which may lead to higher risk, such as hemodynamic instability and arrhythmia [83]. Moreover, the FFR measurement may increase the possibility of complications of the operation [66]. Together, these data suggest that FFR-guided stenting does not show a consistent advantage on prognosis of ACS patients. The solution remains controversial and warrants more rigorous randomized controlled large-scale clinical trials for validation.

The clinical application of FFR is limited by its invasive and time-consuming nature. Among other limitations, some patients are intolerant of adenosine, and the results are easily affected by microcirculation conditions. As a result, new technologies such as transient waveform-free ratio (iFR), coronary angiography flow reserve fraction (CT-FFR), contrast agent-based FFR (QFR), OCT-based FFR (OFR) and IVUS-based FFR (UFR) are emerging as substitutes for FFR. Most of the new techniques have been proven to have good accuracy and reproducibility compared to FFR [84, 85, 86, 87, 88, 89, 90]. Among them, CT-FFR, though noninvasive and superior to CTA findings, is less accurate than FFR in the ACS population [90]. Interestingly, compared with FFR, iFR may even better reflect the actual state of epicardial blood flow in some cases, and the superiority of iFR has been confirmed in some literature [78]. Moreover, iFR does not require adenosine. Some studies demonstrates that the coronary physiological index, which is almost unaffected compared with FFR by microcirculation function, is obtained by measuring the pressure changes on both sides of the lesion during the wave-free period of diastole [91]. However, it has also been suggested that in the acute phase, iFR may be subject to errors such as overestimation of the severity of non-culprit lesions due to increased resting coronary blood flow during ACS [91, 92]. In the 2018 ESC/EACTS guidelines, FFR and iFR are both recommended for evaluating moderate stenotic lesions (Class IA), when evidence of ischemia is not available [3].

In addition to the hemodynamics of the epicardial vessels, microcirculatory resistance is also an important indicator of prognosis [93, 94]. As mentioned previously, IMR is an invasive indicator of the minimum achievable myocardial microvascular resistance. Several studies have demonstrated that IMR values assessed at the time of primary PCI are strongly associated with microvascular obstruction (MVO) [95] and poor prognosis, mainly due to heart failure and malignant arrhythmias [93]. The OxAMI-PICSO study redistributed blood from distant non-ischemic myocardium to the ischemic zone by using pressure-controlled intermittent coronary sinus occlusion (PICSO) patients with IMR >40 prior to stent placement with periodic balloon inflation in the coronary sinus. The results showed the final infarct size is smaller in the intervention group, providing preliminary evidence of the feasibility of using intraoperative IMR guidance for adjuvant therapy in the STEMI population [96]. Remaining trials related to the use of IMR to guide treatment are ongoing, such as a large, randomized trial evaluating the feasibility of delayed stent placement in STEMI patients by exploring the use of IMR values (NCT03581513). Other ongoing research includes the exploration of potential drug treatment options (NCT02894138 and NCT03998319).

## 4. Optimization of Risk Stratification by Combining Multimodal Diagnostic Tools 

The studies discussed above show that it is not sufficient to guide the revascularization of ACS patients only via intracoronary imaging or coronary physiological tools. Currently, a series of experiments combining plaque vulnerability characteristics and coronary physiology is being carried out to improve the prognostic risk classification of different patients. In the ABSORB study, researchers measured the intracoronary NIRS-IVUS imaging of three vessels in patients with myocardial infarction after PCI and implanted stents for vulnerable plaques IVUS-PB ≥65% without blood flow restriction. Results showed that stent treatment for large lipid plaques is safe and effective, significantly increasing the MLA of vessel and long-term positive outcomes [97].

The COMBINE (OCT-FFR) study is a large-scale multicenter prospective, double-blind international study consisting of 547 patients also diagnosed with diabetes. In this trial, culprit plaque and plaques with severe visual estimated stenosis by angiography of ACS patients have been revascularized before. The results showed that although some non-culprit lesions (40%–80% obstruction during angiography by visual examination) were FFR negative (FFR <0.8), the incidence of target lesion-related MACE in TCFA patients after one year was higher than that in patients without TCFA. The researchers’ conclusion did not change when analyzing ACS patients alone [98]. The results confirm that the combination of FFR and OCT can improve the accuracy of identifying vulnerable non-culprit lesions. However, the main population discussed in this study is the diabetic population. Whether their conclusion can be generalized to the general ACS population remains to be studied.

To further validate this idea, an ongoing prospective study (NCT03857971) used both FFR and OCT in 439 patients with ACS to identify the potential impact of preventive PCI in non-culprit lesions with vulnerable traits in the absence of flow restriction on patient prognosis and to prove the necessity of this treatment strategy [99]. As mentioned above, because of the time-consuming and financial burden of combining FFR measurement with other intracoronary imaging tools, using some emerging technologies (such as OFR and UFR) in combination with OCT and IVUS to guide PCI treatment may avoid the potential complexity associated with FFR operation while improving its accuracy. The representative studies are listed in Table 2 (Ref. [97, 98, 99]).

**Table 2.** **The summary of studies for both coronary physiology and intracoronary imaging-guided PCI**.

	People	Number	Technology	Follow up time	Result	Reference
PROSPECT ABSORB	ACS	182	FFR/iFR IVUS	25 months	PCI of angiographically mild lesions was safe and substantially enlarged the follow-up MLA.	[97]
COMBINE	Diabetic mellitus patients with SAP or ACS	547	OCT FFR	18 months	Patients with ≥1 FFR-negative lesions, TCFA-positive patients represented 25% of this population and were associated with a five-fold higher rate of MACE.	[98]
PECTUS-obs	STEMI and NSTEMI	439	OCT FFR	/	ongoing	[99]

ACS, Acute Coronary Syndrome; SAP, Stable Angina Pectoris; MLA, Minimum Lumen Area; MACE, Major Adverse Cardiovascular Events.

QFR, OFR, and UFR are coronary function indexes calculated based on angiography as well as OCT and IVUS images; they demonstrate accuracy and repeatability. Compared with FFR, these technology may also reduce the secondary guidewire’s potential damage and additional costs. Currently, QFR has been widely accepted, and its safety and superiority have been confirmed [100]. The accuracy of QFR compared to FFR was also confirmed in the ACS population [87, 88]. Studies have also confirmed the predictive role of QFR in the prognosis of ACS patients and recommended it as a new tool for risk stratification and therapeutic management [101, 102]. OFR with OCT high-resolution images is better than QFR with traditional images, as it is less affected by the original scaffold [103]. When FFR ≤0.8 is used as the cutoff value to define blood flow restriction, the overall diagnostic accuracy of OFR is 90%, and the sensitivity and specificity are 87% and 92%, respectively [104]. Since OCT is recommended for use in ACS patients to evaluate coronary lesions [3], OFR may more effectively evaluate non-culprit lesions and help realize the concept of complete physiological revascularization. The accuracy of UFR has recently been verified [86]. Good diagnostic accuracy and low observer variability may improve its effectiveness. One such study has already demonstrated that OFR combined with plaque features can help identify high-risk non-culprit lesions [105]. However, as OFR or UFR measurement in China has not been fully automated, its accuracy still needs to be confirmed in more extensive prospective studies. As yet, no clinical trials have applied these techniques to guide patient treatment in clinical settings. Future investigations and clinical applications of imaging-based coronary function indices still need to be explored.

## 5. Summary

In the past ten years, intracoronary imaging technology has guided and optimized the diagnosis and treatment of ACS patients. Concurrently, use of coronary physiological tools has enriched our perspective in evaluating coronary lesions, providing new insights into the pathogenesis of atherosclerosis and the pathophysiology of ACS. These tools have further improved treatment strategy and prognosis. Until now, coronary intervention technology has moved from the original ‘one size fits all’ modality to a new era of ‘precise intervention’. The combination of the two technologies may improve the prognostic stratification of patients, while the generation of FFR substitutes (such as OFR and UFR) can avoid additional harm to patients due to the operation of FFR measurement. It is promising that the integrated assessment of both techniques will be clinically essential to improve the risk stratification of ACS patients and optimize the treatment process for PCI. Combining this substitute with intracoronary imaging may be a new strategy to guide ACS in the future.
